# Optimising Vaginal Microbiome Profiling for Clinical Translation: A Comparative Assessment of Sample Storage Methods and a Vagina-Specific 16S rRNA Gene Database

**DOI:** 10.3390/microorganisms14010128

**Published:** 2026-01-07

**Authors:** Alishum Ali, Jeffrey A. Keelan, Blagica Penova-Veselinovic, Morten E. Allentoft, Michael Bunce, Claus T. Christophersen

**Affiliations:** 1 Trace and Environmental DNA (TrEnD) Laboratory, School of Molecular Life Sciences, Curtin University, Perth, WA 6102, Australia; alishum.ali@postgrad.curtin.edu.au (A.A.);; 2 Perron Institute for Neurological and Translational Science, Perth, WA 6009, Australia; 3 School of Biomedical Sciences, The University of Western Australia, Perth, WA 6009, Australia; jeff.keelan@uwa.edu.au; 4 Division of Obstetrics & Gynaecology, The University of Western Australia, Perth, WA 6009, Australia; blagica.penova-veselinovic@uwa.edu.au; 5 The Raine Study, The University of Western Australia, Perth, WA 6009, Australia; 6 Section for GeoGenetics, Globe Institute, University of Copenhagen, 1350 Copenhagen, Denmark; 7 OceanOmics, Minderoo Foundation, Perth, WA 6009, Australia; 8 School of Medical and Health Sciences, Edith Cowan University, Perth, WA 6027, Australia

**Keywords:** vaginal microbiome, amplicon sequencing, community state types, 16S rRNA, preterm birth, swab sampling, vaginal database

## Abstract

Vaginal microbiome composition has been linked to risk of preterm birth (PTB), a persistent global health challenge. 16S rRNA microbial profiling has identified specific vaginal community state types (CSTs) that have been associated with PTB risk. Diagnostic profiling requires standardised pre-analytical protocols. We evaluated two storage methods and validated a curated, vagina-specific 16S rRNA gene database (VagDB) to enhance annotation. Paired Copan FLOQ swabs from 22 women at high PTB risk were processed for either (a) dry/immediate freezing or (b) Amies-stabilisation/refrigeration. Amplicon sequence variants were generated via 16S rRNA gene (V4) PCR and Illumina sequencing. We assessed diversity, composition, and community state type (CST) allocation. Amies-stabilised samples yielded significantly higher DNA (*p* = 0.003), but this did not alter species richness, evenness, or community structure. VagDB enhanced species-level resolution. PCoA showed robust clustering by participant and CST (*p* < 0.001), irrespective of storage; CST concordance exceeded 90%. Routinely collected vaginal swabs in stabilisation medium with an 8–72 h refrigeration window yield reliable data, supporting the integration of vaginal microbiome profiling into clinical PTB risk assessment.

## 1. Introduction

The human cervicovaginal microbiome (CVM) plays a critical role in maintaining vaginal health and reproductive success [1]. A balanced CVM, often characterised by low microbial diversity and dominance by *Lactobacillus* species, contributes to a protective acidic environment that inhibits the growth of pathogens and reduces the risk of ascending intrauterine infection during pregnancy. Conversely, vaginal dysbiosis, characterised by a reduction in *Lactobacillus* abundance and an increase in microbial diversity, has been associated with adverse reproductive health outcomes, including increased susceptibility to sexually transmitted infections (STIs), pelvic inflammatory disease, and, importantly, preterm birth (PTB) [2,3].

In seminal work, Ravel and colleagues first established using 16S rRNA profiles that there were five distinct vaginal community state types (CSTs) in a multiethnic cohort of reproductive-age women [2]. These CSTs are broadly characterised by the dominance of specific *Lactobacillus* species (CST-I: *L. crispatus*; CST-II: *L. gasseri*; CST-III: *L. iners*; CST-V: *L. jensenii*) or by a diverse community of primarily anaerobic bacteria (CST-IV), often associated with bacterial vaginosis (BV) [4]. These CST classifications have proven remarkably robust across numerous studies, diverse populations, and varying sequencing methodologies [3,4,5,6,7,8,,9,10,11,12,13,14,15,16].

Recent research has increasingly focused on the role of the CVM in the context of PTB, a leading cause of neonatal morbidity and mortality worldwide, affecting ~5–15% of deliveries depending on socio-geographic location [4,8,14,15,17,18,19,20,21,22]. Studies have consistently shown that the presence of CST-IV, and to a lesser extent CST-III (dominated by *L. iners*), during early pregnancy is associated with an increased risk of PTB [4,23,24,25]. This highlights the potential clinical utility of CST determination as a prognostic tool for identifying pregnancies at high risk of PTB. First-trimester screening offers the opportunity for early intervention and potentially improved outcomes [4,26,27].

Despite the growing appreciation of the importance of the CVM, translating microbiome analysis into diagnostic risk profiling has been hampered by several factors. These include the cost of sequencing-based analyses, lack of capacity for providing rapid results, and technical challenges related to sample collection, storage, and data analysis [28]. Unresolved issues at the critical steps of sample collection and storage can introduce biases and affect the accuracy of microbiome profiling [28,29,30,31,32], and because of low microbial biomass and high host DNA content, vaginal samples are particularly susceptible to such biases [20,33,34,35]. Furthermore, the lack of comprehensive, vagina-specific reference databases has historically limited the accuracy of genetic taxonomic assignments, hindering species-level resolution [3].

To mitigate such biases, best-practice recommendations often advocate for immediate freezing of cervicovaginal swabs at −80 °C after collection, prior to DNA extraction and sequencing [29,30]. However, this requirement presents significant logistical challenges for large-scale clinical implementation and epidemiological studies, particularly in resource-limited settings and/or when samples are collected at home or in remote locations. Commercially available stabilisation media, such as the Copan ESwab system with Amies transport medium, offer a potential solution. These systems are designed to preserve bacterial viability while inhibiting overgrowth or lysis, allowing for storage at refrigerated temperatures (4 °C) for several days prior to sample processing [30,36,37].

Previous studies have investigated the impact of sample storage conditions on the vaginal microbiome, but with some limitations. Bai and colleagues compared immediately processed ESwabs to those frozen for 4–6 weeks, but did not compare preservation medium to a dry, immediate-freeze condition [37]. Mattei and colleagues compared DNA extraction methods from ESwabs but did not assess the impact of storage in the preservation medium itself [35]. Thus, a crucial knowledge gap remains: the direct comparability between immediate freezing of dry swabs and preservation in medium.

A further unresolved issue in vaginal microbiome research is the accuracy when relying on generalist 16S rRNA gene databases for taxonomic assignment. Databases such as Greengenes, Silva, and even the Ribosomal Database Project (RDP) are comprehensive but often lack the resolution and specificity required to accurately identify key vaginal bacterial species, particularly within the complex *Lactobacillus* genus, but also amongst anaerobic taxa commonly associated with vaginal dysbiosis [38,39,40,41]. Many vaginal bacteria are closely related, sharing high degrees of 16S rRNA gene sequence similarity, making differentiation based on short hypervariable regions (like V4) challenging [42,43]. This can lead to misclassification or assignment to higher taxonomic levels (e.g., genus or family) rather than the species level, hindering our ability to fully understand the functional roles of specific bacteria in health and disease [11,44,45,46].

Recent efforts, such as the Vaginal Microbial Genome Collection (VMGC) and VIRGO projects, have focused on developing curated, environment-specific databases to improve taxonomic resolution in vaginal microbiome studies [13,44]. They have developed a more comprehensive gene catalogue and reference genomes, significantly improving the accuracy of taxonomic and functional profiling in metagenomic studies. In this context, shotgun metagenomics approaches have been increasingly used as they can overcome some of the limitations of 16S rRNA gene sequencing [47]. This approach, even in shallow sequencing depth, allows for a more accurate identification of vaginal bacteria for better understanding of the functional potential of the microbiota [48]. However, it remains cost-prohibitive, computationally demanding and time-consuming, making it unlikely to be adopted clinically in the short term [31,47,49].

The present study was undertaken to directly compare the impact of sample stabilisation in Amies medium (with 72 h refrigeration) versus immediate freezing (dry swab) on the determination of vaginal community composition using 16S rRNA gene sequencing. The primary aim was to provide practical clinical and research guidance on optimal sample collection and preservation methods for vaginal microbiome studies. A secondary aim was to develop and explore the efficacy of a curated, vagina-specific bacterial species database to improve 16S rRNA taxonomic assignments, particularly for indicator species relevant to PTB risk.

## 2. Materials and Methods

### 2.1. Participants

Twenty-two women were randomly selected from a larger cohort of 244 participants enrolled in the Western Australian Pregnancy Biobank (WAPB) at King Edward Memorial Hospital (KEMH) in Perth, Western Australia. The WAPB is part of a multi-omics characterisation study of pregnancies at high risk of PTB. All participating women provided written informed consent, and the study was approved by the Women and Newborn Health Service Human Research Ethics Committee (EC00350) (RGS0000000705) and the Curtin University Human Research Ethics Committee (HRE2018-0071).

### 2.2. Sample Collection

**Figure 1:**
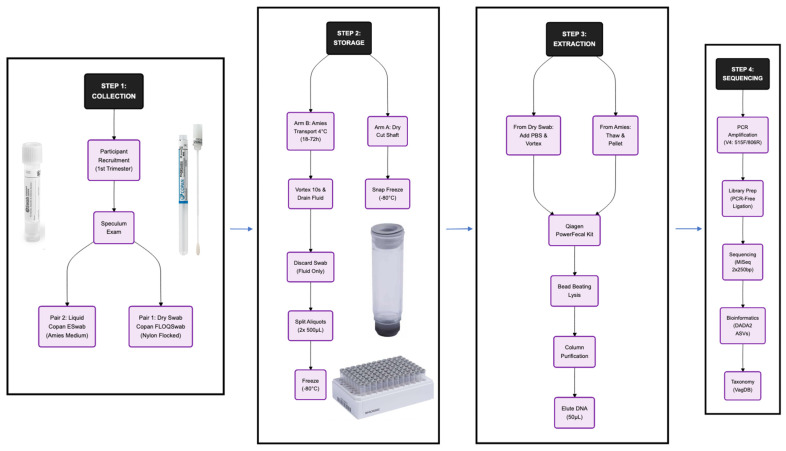
Study workflow and sample stabilisation pipeline. Step 1: Speculum-assisted two sampling methods; (Arm A) a dry exocervical swab immediately stored at −80 °C and (Arm B) an ESwab stored in 1mL Aimes stabilization medium at 4 °C for up to 72 h prior to processing and long-term storage at −80 °C. Step 2 highlights our standard operating procedure for the Aimes arm, involving vortexing and drainage to maximise sample recovery, followed by aliquoting into two 450 µL samples. Step 3 shows synchronized DNA extraction. Step 4 shows our PCR amplification, library preparation, sequencing and bioinformatic analysis.

Cervicovaginal (CV) swabs were collected by a trained WAPB research midwife using a speculum during a routine visit to the KEMH Preterm Birth prevention clinic in the first trimester (9–16 weeks’ gestation). Speculum-assisted collection was employed to minimise potential contamination from the vulva and to ensure consistent sampling of the exocervix. Two pairs of exocervical Copan FLOQSwabs (Copan Diagnostics, Murrieta, CA, USA; Product #520CS01) were obtained simultaneously from each participant. One pair of swabs, designated as “dry and immediate” (DI), was immediately snapped off at the 30 mm breakpoint fit into sterile 0.75 mL internal thread cryotubes (Micronic, Lelystad, The Netherlands; Product #52536-X01). These tubes were then immediately transferred to a −80 °C freezer for long-term storage. The second pair of swabs, designated as “stabilised and refrigerated” (SR), was immediately placed into the provided Copan ESwab tubes containing 1 mL of Amies transport medium (Copan Diagnostics, Murrieta, CA, USA; Product #480C). The swabs were vortexed vigorously to release the collected material into the medium. The Amies medium was then split into two equal aliquots (approximately 450 µL each) and transferred to 0.75 mL internal thread cryotubes. These aliquots were refrigerated at 4 °C for up to 72 h before being transferred to long-term storage at −80 °C (Figure 1).

### 2.3. DNA Extraction

A total of 44 CV swabs (22 DI and 22 SR, matched to the 22 study participants) were retrieved from −80 °C storage for DNA extraction. For the DI swabs, 700 µL of sterile phosphate-buffered saline (PBS) was added directly to the cryotubes containing the frozen swab tips. The tubes were vortexed vigorously to dislodge biological material from the swabs prior to extraction and processing.

For both DI and SR samples, an unused sterile swab was processed alongside as an extraction control. This control swab was either washed with PBS (for DI) or Amies fluid (for SR). All samples (including controls) were then centrifuged at 13,000 RCF for 5 min to pellet the cellular material. DNA was extracted from the pelleted material using the PowerFecal DNA Isolation Kit (QIAGEN, Hilden, Germany) on a QIACube automated platform (QIAGEN), following the manufacturer’s instructions with minor modifications. Briefly, the resuspended pellets were subjected to mechanical lysis using a TissueLyser II (QIAGEN) at 30 cycles/min for 5 min, repeated twice with inversion of the sample between cycles. DNA was eluted in approximately 80 µL of elution buffer. DNA concentrations were quantified using a Qubit 3.0 Fluorometer (ThermoFisher Scientific, Perth, Australia) with the Qubit dsDNA HS Assay Kit. DNA extracts were also assessed for PCR inhibitors (see below) and stored at −20 °C until library preparation.

### 2.4. qPCR Screening

To assess the relative amount of amplifiable bacterial DNA and to detect the presence of PCR inhibitors, a real-time quantitative PCR (qPCR) assay was performed on all DNA extracts. The qPCR targeted the V4 hypervariable region of the 16S rRNA gene, using the broadly conserved primers recommended by the Earth Microbiome Project (EMP): 515F (5′-GTGCCAGCMGCCGCGGTAA-3′) and 806R (5′-GGACTACHVGGGTWTCTAAT-3′) [41,42].

qPCR reactions were carried out in 25 µL volumes, containing: 2 mM MgCl2 (Applied Biosystems Inc. [ABI], Foster City, CA, USA), 4 U/µL AmpliTaq Gold DNA Polymerase (ABI), 1X Taq polymerase buffer (ABI), 0.4 µM dNTPs (Astral Scientific, Sydney, Australia), 0.1 mg/mL bovine serum albumin (BSA; Fisher Biotec, Perth, Australia), 0.2X SYBR Green (Invitrogen, Carlsbad, CA, USA), 0.4 µM of each primer (Integrated DNA Technologies [IDT], Melbourne, Australia), and 4 µL of DNA template (either undiluted or diluted 1:2).

The qPCR cycling conditions were as follows: initial denaturation at 95 °C for 5 min, followed by 35 cycles of 95 °C for 30 s, 54 °C for 30 s, and 72 °C for 30 s, with a final extension at 72 °C for 10 min. Reactions were performed on a StepOnePlus Real-Time PCR System (ABI). Samples that did not amplify adequately (defined as a cycle threshold [Ct] value greater than 32, which is the lowest Ct value produced by a control sample) were not progressed to library preparation, even if DNA was detectable by Qubit (ThermoFisher Scientific, Perth, Australia).

### 2.5. Amplicon Generation and Sequencing

The EMP V4 primers (515F/806R) were modified to include a unique seven-base pair barcode sequence on the 3′ end of the 806R primer, allowing for post-amplification multiplexing. A combinatorial barcoding approach was used. The same qPCR reaction mixture described above was used. Reactions containing uniquely barcoded reverse primers were run for each sample using the StepOne plus with the same conditions described. Extraction controls, non-template PCR controls (NTCs), and an evenly distributed mock community (MSA-1002, ATCC, Masanass, VA, USA), consisting of 20 bacterial species, were included to evaluate PCR bias and potential contamination.

Samples with robust amplification (Ct < 32) were first pooled into “mini pools” of approximately equal amplicon concentrations (within a 5000 deltaRN range). These mini pools were then purified using a QIAquick PCR Purification Kit (Qiagen) according to the manufacturer’s instructions. The purified, tagged amplicons were eluted in 32 µL of elution buffer (EB) and quantified using a Qubit 3.0 Fluorometer. Finally, the mini pools were combined in equimolar concentrations to create a single library for sequencing.

To minimise PCR amplification bias, a PCR-free library preparation approach was employed. The NxSeq AmpFREE Low DNA Library Kit (Lucigen, Middelton, WI, USA) was used to ligate Illumina P5, P7, and TruSeq sequencing adapters to the barcode-tagged amplicons, following the manufacturer’s instructions. Briefly, after ensuring a minimum of 100 ng of amplified DNA in each sample pool, the ligation reaction was performed. The ligated products were purified using the QIAquick PCR Purification Kit (QIAGEN, Hilden, Germany), eluted in 32 µL of EB, and size-selected using a Pippin Prep 2% agarose gel cassette (Sage Science, Beverly, MA, USA) to remove any residual adapter dimers or non-specifically ligated products. The size-selected amplicon pool was purified again (QIAquick PCR Purification) to remove residual ethidium bromide and eluted in 40 µL of EB. The final library was visualised and quantified using a QIAxcel capillary electrophoresis instrument (Qiagen) and a Qubit 3.0 Fluorometer.

The final amplicon library was sequenced on an Illumina MiSeq platform at the Trace and Environmental DNA Laboratory (TrEnD Lab, Perth, Australia) at Curtin University using paired-end 2 × 250 bp reads with a 500-cycle V2 reagent kit and a standard flow cell, following the manufacturer’s protocols.

### 2.6. Bioinformatics

Preprocessing: Raw FASTQ files were downloaded and demultiplexed using the gotta split command from the SHI7 package [50]. Demultiplexed FASTQ files were assessed for quality and integrity using the FASTQC tool, as implemented in the fastqc r package version 0.1.3. After manual inspection and quality assessment, the FASTQ files were further processed to remove all introduced non-biological sequences, including ligated adapters, barcodes, and primers. This was achieved using Cutadapt version 2.10 [51], with all possible primer sequence variants (including those with ambiguous base calls) provided as input. Cutadapt was configured to search for matches at both the 5′ and 3′ ends of the reads. Then, primer-free reads were processed using the DADA2 pipeline (version 1.18) in R (version 4.1.0) to generate an amplicon sequence variant (ASV) table [28,52], refer to Supplementary Table S1. The ASV table was further processed by removing any environmental and reagent contaminants after robust identification using the “isContaminant” function and the “auto” flag from the R package decontam (v1.14.0). This method identifies contaminating bacteria in the samples by testing for an inverse relationship between the ASV abundance and DNA concentration. It further assesses if the ASV is enriched in the negative controls (extraction and PCR negative). Any ASVs identified as a contaminant above the threshold of 10% were permanently removed from the table. As part of our decontamination process, we removed all control samples from the dataset, including the Mock community sample and then removed any ASVs that remained at 0 abundance.

Vaginal-Specific Database Development: To improve the accuracy of taxonomic assignments, we developed a curated, vagina-specific 16S rRNA gene database. To construct this database, referred to as VagDB, we leveraged the Genome Taxonomy Database release 207 (GTDBr207), a comprehensive and phylogenetically robust resource, as our primary source of reference sequences [53], which we have further formatted and made available publicly to be used for the DADA2 pipeline database found here https://zenodo.org/records/6655692 (accessed on 17 June 2022). The VagDB construction began by compiling a list of 582 bacterial species previously reported to be present in the human vagina [3]. Using this species list, we extracted the corresponding 16S rRNA gene sequences from the GTDBr207 database. The extracted sequences were formatted to be compatible with the DADA2 R package version 1.26.0 assignTaxonomy and addSpecies functions. This involved creating a FASTA file containing the sequences and a separate taxonomy file mapping each sequence to its corresponding taxonomic lineage (Kingdom, Phylum, Class, Order, Family, Genus, Species). We used Silva database release 138 to cross-reference taxonomy assignations, employing the GTDB taxonomic nomenclature. The VagDB database is publicly available at https://zenodo.org/records/17452627 (accessed on 27 October 2025).

Mock Community Analysis: To assess the robustness of our PCR workflows, we included a mock community sample. The resulting ASV generated through our bioinformatic pipeline was searched against the known composition of the ATCC 1002 20 species mock community [54]. A DADA2 formatted GTDB extracted 16S rRNA sequences can be found here https://zenodo.org/records/4781067 (accessed on 22 May 2021).

CST Profile Determination and Allocation: Vaginal community state types (CSTs) were assigned to each sample using the VALENCIA (VAginal Community State Type Nearest Centroid ClassifiEr) method. VALENCIA is a nearest-centroid classifier that assigns samples to predefined CSTs based on their overall taxonomic composition. We used the updated VALENCIA reference database, which includes 13 sub-CSTs, providing finer-grained resolution than the original five CSTs defined by Ravel et al. [2,55] (Supplementary Table S2).

### 2.7. Statistical Analysis

All data exploration, statistical analyses, and visualisations were performed primarily using R (version 4.1.0) and the phyloseq package (version 1.38.0) [56]. A reproducible R Markdown document containing the complete analysis pipeline is available (Supplementary File). The decontam R package (version 1.14.0) was used to identify and remove potential contaminant ASVs, using the “combined” method, which considers both the frequency of ASVs in samples and their prevalence in negative controls [57].

Alpha diversity metrics (Shannon diversity and phylogenetic diversity) were calculated at the ASV level using the vegan (version 2.5-7) and picante (version 1.8.2) R packages. Prior to alpha diversity calculations, ASVs with zero counts across all samples were removed. Differences in alpha diversity between the two storage conditions (DI and SR) were assessed using paired Wilcoxon signed-rank tests, with Bonferroni correction for multiple comparisons. The ggstatsplot R package (version 0.9.0) was used for statistical testing and visualisation [58].

Beta diversity, reflecting the differences in community composition between samples, was calculated using the Bray–Curtis dissimilarity metric on species-level, proportion-normalised data. Principal Coordinates Analysis (PCoA) was performed using the phyloseq package to visualise the beta diversity patterns. Permutational Multivariate Analysis of Variance (PERMANOVA) using the adonis function in the vegan package was used to test for significant differences in community composition between storage conditions, with 999 permutations.

## 3. Results

### 3.1. Impact of Storage Condition on DNA Yield and PCR Amplification

A total of 48 samples were sequenced, including 44 vaginal swabs (22 DI and 22 AS), two extraction blanks (one for each storage condition), one non-template PCR control (NTC), and one ATCC mock community sample. All samples, except for the extraction blanks and NTC, had detectable DNA concentrations, defined as a Qubit dsDNA concentration greater than 0.1 ng/µL.

**Figure 2:**
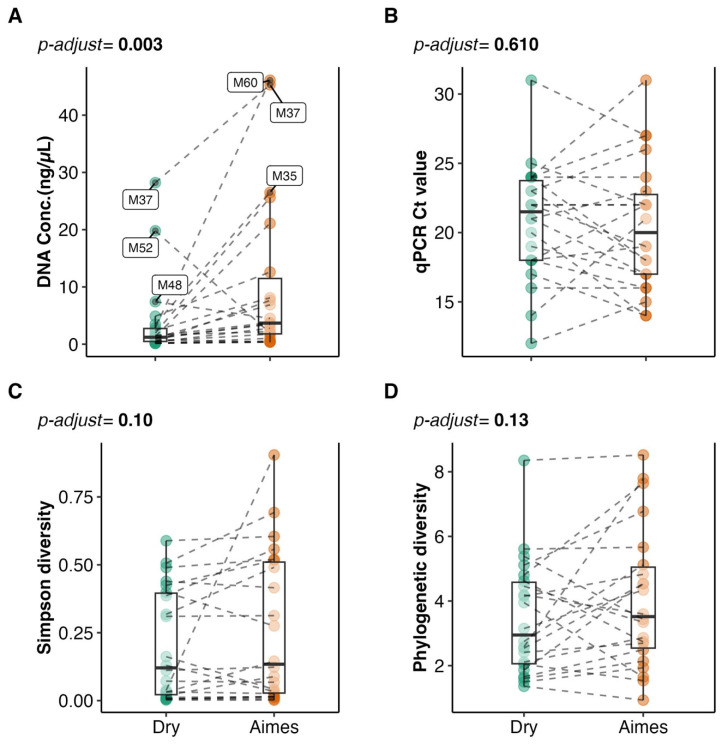
Swab storage condition impacts DNA yield and alpha diversity but not amplification efficiency. (**A**) Boxplot representing DNA concentration (ng/µL) on the y-axis and storage condition on the x-axis. (**B**) Boxplot comparing qPCR Ct values between storage groups. (**C**) Simpson alpha diversity measuring impact of storage conditions on species richness and evenness of the sample. (**D**) Within-sample diversity measured according to Faith’s phylogenetic distance, measured by the total branch length between species in the tree. Each point represents a patient sample with the colour indicating storage condition. Dotted lines link paired samples from the same patient. A paired Wilcoxon ranked sum test is used to compare means. Significance is assumed if Bonferroni-adjusted *p* < 0.05.

As shown in Figure 2A, the mean DNA concentration was significantly higher in the AS samples compared to the DI samples (*p* = 0.003, paired Wilcoxon signed-rank test). This difference is likely attributable to the more efficient release of biological material from the swab tip in the liquid Amies medium compared to the dry-frozen swab, where material may have adhered more strongly to the swab tip.

Despite the difference in DNA yield, all barcoded samples, except for the extraction blanks and NTC, amplified successfully during the initial qPCR screening (Figure 2B). Ct values were not significantly different between the DI and AS groups (*p* = 0.610, paired Wilcoxon signed-rank test), indicating that the storage condition did not substantially impact PCR amplification efficiency.

### 3.2. Sample Read Counts and Alpha Diversity Comparisons

After demultiplexing, a total of 4,705,194 reads were obtained across the 48 samples. Following quality filtering, processing, and removal of reads from the extraction blanks and NTC, 81% of the initial reads (3,814,012) remained. The minimum number of reads per sample was 19,836, the maximum was 255,012, and the mean was 86,682. These reads were distributed across 330 ASVs, representing putative bacterial species. Approximately 13% of the ASVs (represented by only 63 reads) were identified as singletons or doubletons. ASVs that were preferentially detected in the negative controls and clearly not of human vaginal origin were identified as contaminants and removed from the dataset prior to downstream analyses. Analysis of the mock community sample revealed that 19 out of the 20 expected species were successfully detected, demonstrating the sensitivity of the PCR and sequencing approach (Supplementary Figure S1).

Despite the significant difference in DNA yield between the DI and AS samples, there were no statistically significant differences in alpha diversity metrics (Shannon diversity and phylogenetic diversity) between the two storage groups (*p* ≥ 0.01, paired Wilcoxon signed-rank tests with Bonferroni correction). Figure 2C shows the Simpson alpha diversity index, and Figure 2D shows Faith’s phylogenetic diversity index. Although not statistically significant, a trend towards slightly greater richness in the AS group was observed.

### 3.3. Microbiota Composition and CST Accuracy

**Figure 3:**
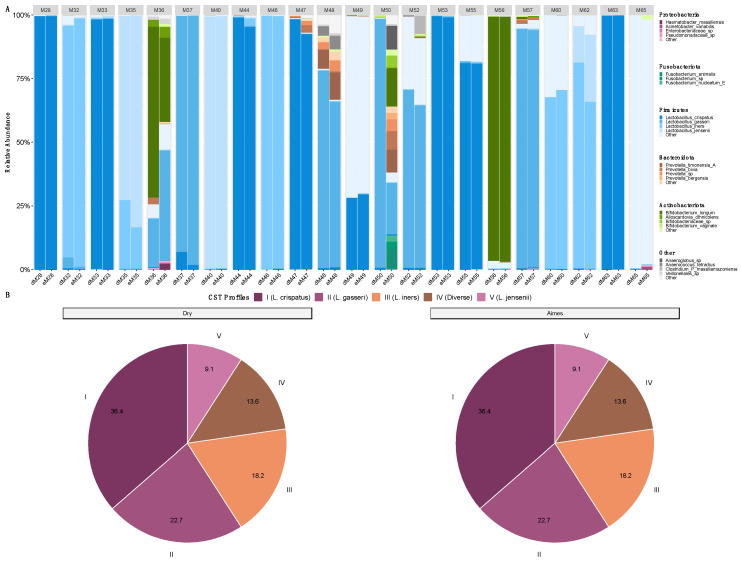
Relative abundance of top bacterial species and Cohort CST breakdown. (**A**) Stacked bar chart is organised by participant number; sample category is denoted on the x-axis (letter d or e in front of patient id is to denote Dry or Aimes group, respectively). Y-axis is the relative abundance from 0–100%. Bars are coloured according to species composition. (**B**) Pie chart representing CST composition according to storage type. Segments are coloured by CST type. External labels show CST name and internal values are percentage of all CST profiles.

Overall, there was minimal impact of storage condition (DI vs. AS) on the detection and relative abundance of vaginal bacterial species commonly found in pregnant women. The relative abundance profiles for each sample pair (DI and AS from the same woman) are shown in Figure 3A. In most cases, the relative abundance of major bacterial taxa was comparable and consistent between the two storage conditions. However, for two participants (M36 and M50), the relative abundance profiles showed some discrepancies between the DI and AS samples.

CST allocation was 91% similar between the DI and AS samples. For all samples, *L. crispatus* (CST-I) dominated in 36.4%, *L. gasseri* (CST-II) in 22.7%, *L. iners* (CST-III) in 18.2%, anaerobic bacteria or high alpha-diversity (CST-IV) in 13.6%, and *L. jensenii* (CST-V) in 9.1%. However, for the two discordant samples (M36 and M50), these were assigned conflicting CSTs. For participant M36, the DI sample was classified as CST-IV with an 89% confidence score, while the AS sample was classified as CST-II with a 64% confidence score. For participant M50, the opposite pattern was observed: the DI sample was classified as CST-II with an 84% confidence score, while the AS sample was classified as CST-IV with a 33% confidence score.

**Figure 4:**
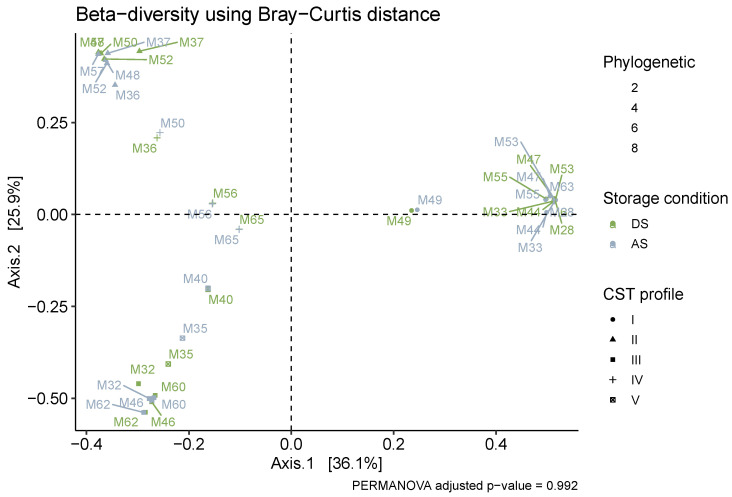
Beta-diversity calculated based on the Bray–Curtis dissimilarity metric on proportion-normalized counts. Most inter-sample compositional variation was explained by the first 2 dimensions that were visualized on the PCoA plot. Samples are coloured by storage condition, diversity indicated by point size and CSTs are indicated by symbol type. FDR-adjusted statistical significance (*p* < 0.05) was tested using Wilcoxon for alpha-diversity and PERMANOVA stratified on patient ID for beta-diversity.

Beta diversity analysis, expressed as the Bray–Curtis dissimilarity and visualised with PCoA (Figure 4), showed no distinct clustering of samples based on storage condition. Instead, samples clustered strongly by participant ID at the individual level and by CST at the broader community level. This indicates that the inter-individual differences in vaginal microbiome composition were far greater than any differences introduced by the storage method. PERMANOVA analysis confirmed the lack of a significant effect of storage condition on overall community composition (*p* = 0.992). However, as expected, CST was a highly significant predictor of community composition (R^2^ = 0.81, *p* < 0.0001). While the DI and AS samples from participants M36 and M50 showed some separation on the PCoA plot, this occurs within the context of principal coordinates 1 and 2, explaining 62% of total variation. This separation is explained largely by Axis 1 (36.1% of variation) and a large but reduced explanation by Axis 2 (25.9%). While some differences are observable between storage conditions in these two patients, they are insignificant in explaining the overall sample variance compared to the substantial community differences that are brought about by CST’s community structure.

## 4. Discussion

The relationship between the cervicovaginal microbiome (CVM) and reproductive health outcomes is increasingly recognised, with implications for conditions ranging from sexually transmitted infections [59] and cancer [60] to conception [61] and preterm birth [62]. Specifically, a shift in CVM composition during early pregnancy from a low-diversity, *Lactobacillus*-dominated state to a high-diversity, dysbiotic state is a well-established risk factor for adverse outcomes [22,23,47,63,64].

Despite the growing body of evidence, CVM analysis has not yet been widely adopted in routine clinical practice. This is partly due to the cost and technical complexities associated with microbiome sequencing, as well as the challenges of standardisation and minimising bias at various stages of the workflow, from sample collection and storage to DNA extraction, PCR amplification, and bioinformatics analysis [28]. Recent literature has highlighted the cumulative nature of microbiome bias, with upstream steps, such as sample storage, potentially having a significant impact on downstream results [35]. This is particularly relevant for low-biomass samples, such as cervicovaginal swabs, where even small variations in sample handling can introduce substantial bias. Furthermore, accurate species-level taxonomic identification, crucial for understanding the functional roles of different vaginal bacteria, remains a challenge due to limitations in commonly used 16S rRNA gene sequence databases [3,13].

Routine collection of CV swabs for microbiome studies would be significantly more practical and economical if simple dry swabs could be collected and stored at readily accessible temperatures, such as those found in standard household freezers [65,66]. The primary aim of this study was to determine whether CV samples collected in Amies stabilisation medium and refrigerated for up to 72 h yield comparable vaginal DNA yields and microbiome profiles to those obtained from dry swabs immediately frozen at −80 °C.

Our results demonstrated that while DNA yield was significantly higher in the Amies-stabilised swabs (*p* < 0.003), the difference did not significantly influence the relative abundance of bacterial taxa, the overall community composition (beta diversity), or the assignment of samples to CSTs. This result suggests that the Amies medium effectively preserves the integrity of the vaginal microbiome profile, even with a delay in freezing. The higher DNA yield in the Amies samples is likely due to the more efficient release of cells and DNA from the swab tip in the liquid medium compared to the dry-frozen swabs. The difficulty of dislodging biological material from the dry swab, requiring PBS solution, highlighted deficiencies in the method.

Although we observed some discrepancies in CST allocation for two sample pairs (M36 and M50), these differences were relatively minor and likely attributable to low DNA yield and potential amplification bias in the DI samples [28,35,67,68]. Importantly, beta-diversity analysis showed that these samples still clustered more closely with each other and with other samples from the same CST than with samples from different CSTs, indicating that the overall community structure was preserved.

While most CVM profiles in our cohort fell within the expected common CSTs, one participant (M65) was assigned to CST-IV despite having a community dominated by *Lactobacillus*. Further analysis revealed that this sample was classified as sub-CST IV-B, which is characterised by a moderate abundance of anaerobic bacteria, such as *Bifidobacterium vaginale* (AKA *Gardnerella vaginalis*) and *Fannyhessea vaginae* (AKA *Atopobium vaginae*), alongside *Lactobacillus*. This highlights the importance of using refined CST classifications and accurate taxonomic assignments to distinguish between potentially different functional states within the broader CST categories [13,55].

### 4.1. Strengths and Limitations

A key strength of this study is its direct comparison of two commonly used sample storage methods (immediate dry freezing vs. stabilisation medium with refrigeration) under conditions that are relevant to both clinical and research settings. It addresses the gap in the literature with a direct comparison and demonstrates the accuracy. To our knowledge, only one other study, by Bai et al., has examined the impact of vaginal sample storage conditions [37]. While their work was valuable, it focused on a comparison between immediately processed ESwabs and frozen ESwabs, and did not include a direct comparison to dry, immediately frozen swabs; thus, it cannot deduce any storage method differences.

Another strength is our use of a rigorous bioinformatics pipeline, including single-round PCR amplification followed by adapter ligation and paired-end sequencing, to minimise amplification bias, tag jumping and contamination [69]. We also developed and implemented a curated, vagina-specific 16S rRNA gene database (VagDB) to improve the accuracy of taxonomic assignments, addressing a critical limitation of previous studies that relied on more generalist databases [44]. The identification of key genotypes for the diagnosis of CST could open the door for more rapid point-of-need sample processing and detection.

However, this study also has limitations. We did not compare our samples to freshly collected samples (i.e., those processed immediately without any storage). While this would have provided an absolute “gold standard,” it was not logistically feasible within the context of this study. Our swabs were stored for a significant period (6–12 months) at −80 °C, which may have introduced some changes, although previous studies suggest that long-term storage at −80 °C is generally effective for preserving microbiome profiles [29,30]. Another limitation is the relatively small sample size (*n* = 22), which may have limited our power to detect subtle differences between storage conditions, particularly within specific subgroups. Finally, while we used a rigorous bioinformatics pipeline and a curated database, taxonomic assignments based on 16S rRNA gene sequencing, particularly the V4 region, still have inherent limitations in resolving closely related species—a limitation that could potentially be resolved using longer read and/or shotgun approaches, as we have shown in other works [47,70].

### 4.2. Addressing the Taxonomic Database Issue

A significant challenge in microbiome research, particularly in specialised niches, is the accurate taxonomic assignment of sequence reads. Generalist 16S rRNA gene databases, such as Greengenes, Silva, and RDP, often lack the resolution and specificity required to accurately identify vaginal bacterial species [39]. This is because many vaginal bacteria, particularly within the *Lactobacillus* genus and among anaerobic taxa associated with dysbiosis, are closely related and share high degrees of 16S rRNA gene sequence similarity. This can lead to misclassification or assignment to higher taxonomic levels (e.g., genus or family) instead of the species level, hindering our ability to fully understand the functional roles of specific bacteria.

To address this, we developed VagDB, a curated, vagina-specific 16S rRNA gene database. VagDB was built upon the robust framework of the Genome Taxonomy Database (GTDB) [53], which uses a phylogenomic approach to define bacterial taxonomy, providing a more accurate and stable classification than traditional 16S rRNA gene-based approaches. We extracted 16S rRNA gene sequences from GTDB for 582 bacterial species previously reported to be present in the human vagina [3], creating a curated database that will enhance taxonomic assignment confidence at the species level. We further cross-referenced and validated our taxonomic assignments using a secondary database built upon the Silva 138 release. This dual-database approach enhanced the reliability of our species-level identifications. The use of VagDB, in conjunction with the stringent 100% sequence identity criterion for species assignment in DADA2, significantly improved the resolution and accuracy of our taxonomic assignments, allowing us to distinguish between closely related *Lactobacillus* species and other vaginal taxa. The VagDB database is formatted for compatibility with DADA2 and is freely available.

## 5. Conclusions

In conclusion, our study demonstrates that immediate freezing of dry cervicovaginal swabs is not strictly necessary for accurate vaginal microbiome profiling using 16S rRNA gene sequencing. Collection and storage of swabs in a stabilisation medium, such as Copan ESwab with Amies, followed by refrigeration for up to 72 h and subsequent freezing at −80 °C, yields comparable results. While DNA yield is significantly higher with the stabilisation medium, this does not introduce significant bias in terms of community composition, alpha diversity, beta diversity, or CST allocation.

This finding has important implications for both clinical and research applications of vaginal microbiome analysis. It allows for greater flexibility in sample collection and storage, facilitating the integration of microbiome profiling into routine clinical workflows and large-scale epidemiological studies, including those conducted in resource-limited settings or involving self-collection of samples at home. The ability to refrigerate samples for up to 72 h before freezing provides a practical window for both transport and processing, without compromising the integrity of the microbiome data. Additionally, our development and implementation of a curated, vagina-specific 16S rRNA gene database (VagDB) significantly enhances the accuracy and resolution of taxonomic assignments, enabling more precise characterisation of the vaginal microbiome and a better understanding of the roles of specific bacterial species in health and disease, particularly in the context of preterm birth risk stratification.

While our protocol is robust for amplicon sequencing, the benefits must be balanced against unintentional obstacles created for other downstream applications, such as metagenomics or metatranscriptomicsthat can be caused by a delay in freezing. In our previous work, we demonstrated that metagenomics, even with shallow depth of sequencing, offers better taxonomic resolution across all DNA lifeforms than 16S metabarcoding [47]. Future use of samples in multi-omics studies and especially long-read sequencing must consider the long-term viability of high-molecular-weight DNA; in addition, labile RNA may be better preserved via immediate freezing. Consequently, while sample stabilisation is effective in potentially enabling microbiome integration within clinical practice, immediate freezing may be preferred to maximize nucleic acid integrity for multi-omic studies.

Future research should focus on validating these findings in larger and more diverse populations, including longitudinal studies that track changes in the vaginal microbiome over the course of pregnancy and during the menstrual cycle. Further refinement of the VagDB by incorporating new whole-genome sequence-derived full-length 16S rRNA will improve taxonomic resolution to subspecies levels. Ultimately, the integration of vaginal microbiome analysis into routine clinical practice, facilitated by the removal of rigid cold chain logistics and the design of a rapid and robust laboratory workflow, holds great promise for a range of clinical applications with benefits to women’s health.

## Data Availability

The datasets generated and analysed during the current study are available in the NCBI Sequence Read Archive (SRA) under BioProject accession number PRJNA1348951. The VagDB database is available at https://zenodo.org/records/17452627 (accessed on 27 October 2025).

## Supplementary Materials

The following supporting information can be downloaded at: https://www.mdpi.com/article/10.3390/microorganisms14010128/s1, Table S1: Modified DADA2 Pipeline Parameters for ASV Inference and Taxonomic Assignment; Table S2: Vaginal Community State Type (CST) Classifications and Characteristics; Figure S1: Stacked bar chart showing DNA based ATCC evenly distributed (5%) mock community results.
