# Supplementary material for: Optimising Vaginal Microbiome Profiling for Clinical Translation: A Comparative Assessment of Sample Storage Methods and a Vagina-Specific 16S rRNA Gene Database
Source: Microorganisms. 2026 Jan 7;14(1):128. doi: 10.3390/microorganisms14010128 (PMC12844235; doi:10.3390/microorganisms14010128)
Supplement: Supplementary file 1 [file microorganisms-14-00128-s001.zip › microorganisms-4040102-supplementary.pdf]

Supplementary Information

ASV Inference and Taxonomic Assignment

The following parameters within the DADA2 pipeline were modified from their default values (Table 2). The DADA2-generated ASV table and taxonomic assignments were further refined. Any ASVs with more than 3 hits at 100% identity in the curated vaginal database (described below) was replaced with "Genus_spp.". If no confident species assignment, then "_sp" was added to the last confidently assigned taxonomic level. A phylogenetic tree, required for downstream diversity analyses, was constructed using the maximum likelihood method with the GTR+G+I (Generalised Time-Reversible with Gamma rate variation and a proportion of invariant sites) model, implemented in the phangorn R package [49].

Supplementary Table S1. Modified DADA2 Pipeline Parameters for ASV Inference and Taxonomic Assignment

Function	Parameter	Value	Purpose/Rationale
filterAndTrim	truncLen	c(225, 230)	Truncate reads to consistent lengths
filterAndTrim	maxEE	c(1, 1)	Filter reads with excessive expected errors
dada	pool	"pseudo"	Enable pseudo-pooling for improved error rate estimation
removeBimeraDenovo	method	"per-sample"	Remove chimeric sequences on a per-sample basis
assignTaxonomy	minBoot	80	Minimum bootstrap support of 80 for confident genus-level taxonomic assignments
addSpecies	matching	100%	100% query to database sequence matching for species assignment

Note: All other parameters were maintained at their default values. The 100% species assignment criteria were rigorously implemented.

Supplementary Table S2. Vaginal Community State Type (CST) Classifications and Characteristics

CST	Sub-CST	Dominant Species/Characteristics	Description
I	I-A	*Lactobacillus crispatus*	High relative abundance of *L. crispatus*
I	I-B	*Lactobacillus crispatus*	Moderate relative abundance of *L. crispatus*
II	-	*Lactobacillus gasseri*	Dominated by *L. gasseri*
III	III-A	*Lactobacillus iners*	High relative abundance of *L. iners*
III	III-B	*Lactobacillus iners*	Moderate relative abundance of *L. iners*
IV	IV-A	Diverse bacterial community	Moderate abundance of BV-associated bacteria
IV	IV-B	Diverse bacterial community	High abundance of BV-associated bacteria
IV	IV-C0	*Prevotella*-dominated	Diverse community with *Prevotella* dominance
IV	IV-C1	*Streptococcus*-dominated	Diverse community with *Streptococcus* dominance
IV	IV-C2	*Enterococcus*-dominated	Diverse community with *Enterococcus* dominance
IV	IV-C3	*Bifidobacterium*-dominated	Diverse community with *Bifidobacterium* dominance
IV	IV-C4	*Staphylococcus*-dominated	Diverse community with *Staphylococcus* dominance
V	-	*Lactobacillus jensenii*	Dominated by *L. jensenii*

Note: CST IV types are characterized by diverse bacterial communities often associated with bacterial vaginosis (BV). Sub-classifications are based on relative abundance of specific bacterial genera or BV-associated bacteria.

Mock Community Analysis

The figure below shows a graphical representation of the mock community analysis. The mock community used in this study consisted of the ATCC MSA-1002, 20 Strain Staggered Mix Genomic Material.

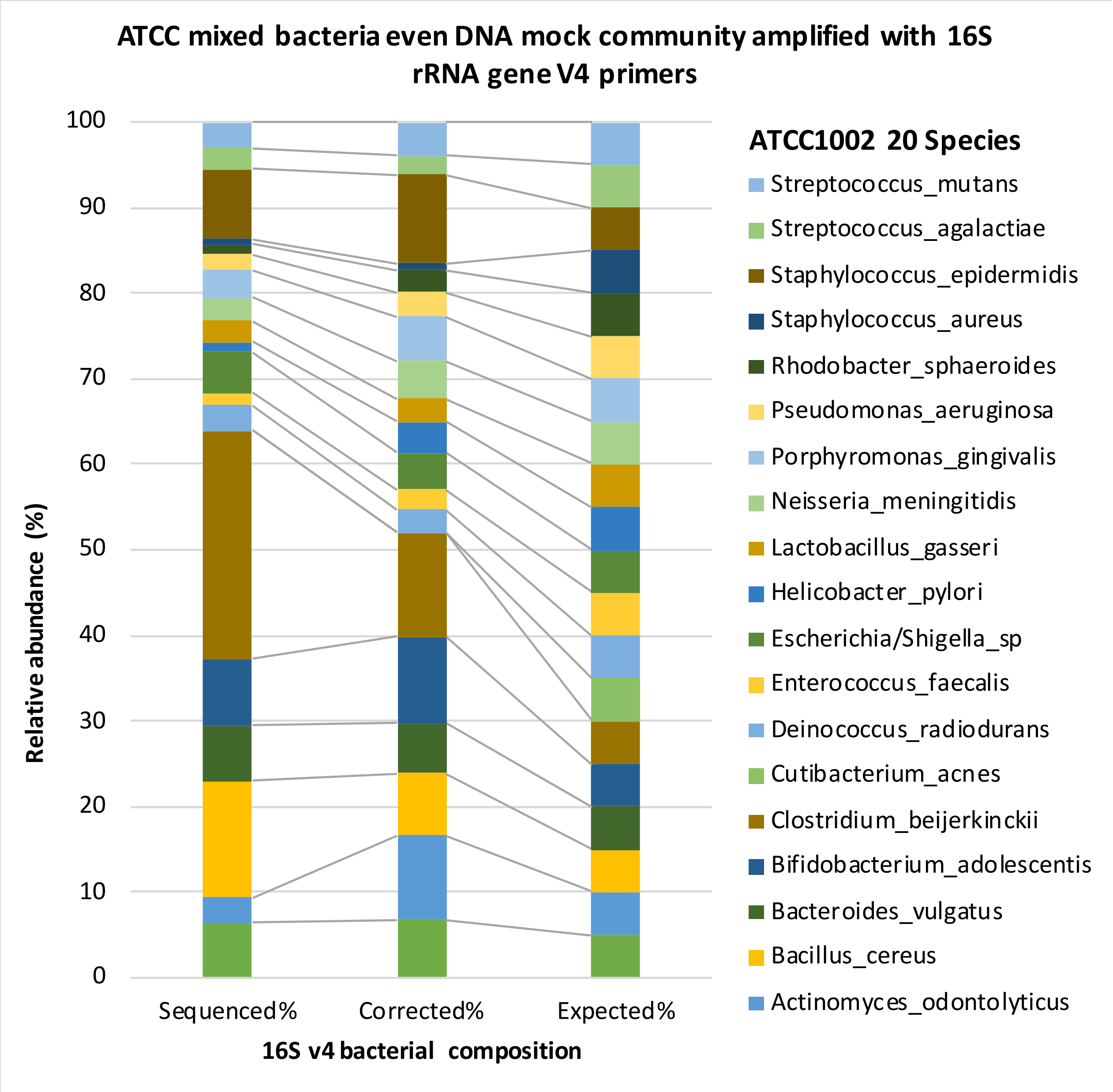

Supplementary Figure S1. Stacked bar chart showing DNA based ATCC evenly distributed (5%) mock community results. On X-axis “Sequenced%” refers to the raw relative abundances of the mock species as detected by our primers, “Corrected%” refers to relative abundance of 16S rRNA gene copy number corrected reads and “Expected%” refers to the DNA percentage of each species in the mock sample as given by ATCC. Y-axis shows the relative abundance measure for reads assigned to each specie of the mock community out of 100%. The colours

represent the different species. Lines link each specie in all three depictions of the mock community.
